# Long-term implications of structured transition of adolescents with inflammatory bowel disease into adult health care: a retrospective study

**DOI:** 10.1186/s12876-019-1046-5

**Published:** 2019-07-19

**Authors:** Lukas Schütz, Michael Radke, Stephan Menzel, Jan Däbritz

**Affiliations:** 1 Department of Paediatrics, University Hospital Rostock, Ernst-Heydemann-Str. 8, D-18057 Rostock, Germany; 2 Department of Paediatrics, Klinikum Westbrandenburg, Potsdam, Germany; 3 Ernst-von-Bergmann Outpatient Clinic, Gastroenterology & Hepatology, Potsdam, Germany

**Keywords:** Paediatrics, Epidemiology, Quality of life, Socio-economical and psychological end points

## Abstract

**Background:**

We aimed to evaluate the long-term clinical and socioeconomic outcome of structured transition care in adolescents with inflammatory bowel disease (IBD).

**Methods:**

We compared the clinical long-term course of 24 patients with and 11 patients without structured transition care within 24 months before and 24 months after transfer from paediatric to adult health care. Socio-economic parameters and quality of life were assessed by IBD Questionnaire (IBDQ-32) and additional items. Treatment costs were calculated for medication, surgery and hospitalisation.

**Results:**

The percentage of transfer group patients with an IBD-related intestinal complication was higher compared to the transition group (64% vs. 21%, *p* = 0.022). We also found a tendency towards a higher number of IBD-related surgery in the transfer group compared to the transition group (46% vs. 13%, *p* = 0.077). Transfer group patients received higher mean cumulated doses of radiation compared with the transition group (4.2 ± 5.3 mSv vs. 0.01 ± 0.01 mSv, *p* = 0.036). Delayed puberty was only noted in the transfer group (27%, *p* = 0.025). Mean expenditures for surgeries and hospitalisation tended to be lower in the transition group compared to transfer group patients (744 ± 630€ vs. 2,691 ± 4,150€, *p* = 0.050). Sexual life satisfaction was significantly higher (*p* = 0.023) and rates of loose bowel movements tended to be lower (*p* = 0.053) in the transition group.

**Conclusions:**

Structured transition of adolescents with IBD from paediatric into adult health care can lead to important clinical and economic benefits.

**Electronic supplementary material:**

The online version of this article (10.1186/s12876-019-1046-5) contains supplementary material, which is available to authorized users.

## Background

Chronic diseases in adolescent patients can be an enormous burden when the time of moving out of their parental home and becoming independent coincide with the first referral to the adult clinic after many years of paediatric health care. Loss of continuity of care at transfer might endanger disease remission. Onset of inflammatory bowel diseases (IBD) appears frequently in young age [1]. The incidence of Crohn’s disease (CD) amounts up to 6.6 per 100,000 people in Germany, while the prevalence is 100 to 200/100,000 people [2]. Ulcerative colitis (UC) affects up to 250 of 100,000 people and up to 4/100,000 people are newly diagnosed each year [3]. About 25% of IBD patients are being diagnosed before the age of 18 years [4]. Thanks to effective modern therapy options, adolescents with IBD can achieve disease remission and a normal life [5]. However, young adults need a different clinical setting than adolescents in order to live independently with their disease. A structured transition program has important benefits and can provide a comprehensive, accompanied transition from adolescent to adult care. This is because deficiencies in care during this particularly critical phase can result in treatment interruptions, insufficient treatment compliance, and increased frequency of complications that are probably avoidable [6]. Research in transition care for IBD patients was long time limited to expert recommendations and experiences. Strengthening the disease management skills of adolescents, while providing information about transition and slowly reducing the parental involvement was described as beneficial by health care providers and patients [7, 8]. However, the definition of an effective transition process is still a matter of intensive debate. The American Association of Paediatrics (AAP) described six core elements as a guidance for transition programs: transition policy, monitoring, readiness and planning, as well as transfer of care and transfer completion [9]. While transition care developed relatively slowly in Germany, three out of four specialist paediatric centres for IBD in the United Kingdom (UK) offered social support for transition already in 2007 [10]. However, structured transition has developed increasingly in the last 10 years in Germany and some initiatives demonstrate promising results. Transition clinics, case managers and a standardised medical summary for the adult gastroenterologist are the main parts of the nationwide Berlin Transition Program (BTP) [11, 12]. In addition, there are successful patient education programs in place, which inform the patient about the program, risky health behavior, sexuality and the search for a new physician within these services [13, 14].

A study from the UK could demonstrate several clinical benefits in IBD patients after visiting a multi-disciplinary transition clinic [15]. A Canadian study, however, failed to find an alteration of acute health care utilisation depending on the post-transfer setting (community based vs. specialised tertiary centre) two years after transfer [16]. Nonetheless, most of the studies evaluating the disease course of IBD after transition did not include economic parameters and only a few investigated the psychosocial outcome [17]. We thus aimed to compare the clinical and socioeconomic long-term outcome in young adults with and without utilisation of a structured transition program at a German hospital.

## Methods

### Patients and study design

We retrospectively identified 52 IBD patients, which were followed up at the Department of Paediatrics of the Klinikum Westbrandenburg (Potsdam, Germany) and afterwards transferred to a nearby gastroenterologist outpatient clinic. Seventeen of them were excluded because they did not meet the inclusion criteria, which required a minimum length of treatment in the paediatric and adult care of at least two years, respectively. Finally, we conducted a retrospective review of medical records of the remaining 35 IBD patients (20 with CD, 13 with UC and 2 patients with unclassified IBD (IBDU)). Included patients were diagnosed with IBD between June 1998 and June 2012 and were transferred to the adult clinic between June 2007 and October 2014. Twenty-four patients had a structured transition (transition group), whereas 11 patients did not receive such transition support (transfer group).

The Klinikum Westbrandenburg implemented a structured transition service for paediatric IBD patients in 2009. The program includes a joint consultation at the age of 18 years before the first visit at the adult clinic with the paediatric and a local adult gastroenterologist, as well as a specialised IBD nurse and the patient. Parents are most commonly not present during these visits. Topics of the visit are disease related knowledge, career plans, medical history, current and further treatment, as well as personal concerns of the patient about the future. The adult specialist is introduced through regular seminars with physicians, patients and their families, when the concept of transition is explained. The paediatric gastroenterologist sends a structured written summary of the previous medical patient history to the adult gastroenterologist immediately after the joint consultation. This summary was developed by the previously mentioned BTP. In addition, the paediatric gastroenterologist empowers the patient to autonomous disease management and provides disease-related knowledge years before the joint transition appointment. This process is guided by a variety of educational milestones recommended by the NASPGHAN checklist for transition [18]. As current data and expert recommendations implied, transition is not only the time of transfer to an adult specialist at the age of 18 years, but also a yearlong process of psychosocial development for patients, parents and physicians that starts much earlier [6, 19]. Therefore, we decided to retrospectively follow up the long-term clinical development of patients during the last two years in paediatric care to the end of their first two years of adult health care. The first follow-up at the adult gastroenterological clinic defines the transfer date.

### Definition of outcome values and data collection

We collected baseline characteristics including demographics, age at diagnosis, disease presentation and medical treatment. Questionnaires included information about socioeconomic parameters such as academic and employment status. The Paris classification defines IBD presentation at diagnosis [20]. We collected data on biological and non-biological treatment of IBD, anthropometric data, intestinal complications (fistula, abscess, stenosis, dysplasia/neoplasia and necessity of bowel resection), the number of hospital admissions and surgeries and the number of endoscopies within the study period. The cumulated radiation exposure was composed of the diagnostic imaging studies during the study period and presented in millisieverts (mSv). Pyoderma gangrenosum, erythema nodosum, peripheral arthritis, axial arthropathy, primary sclerosing cholangitis, uveitis, psoriasis and aphthous stomatitis were considered as extraintestinal manifestations (EIM) of IBD [21, 22]. Newly diagnosed psychiatric comorbidities (depressive, anxiety, adaptive and eating disorder, disordered eating) were documented. We defined haematopoietic disorder, polyneuropathy, dermatological adverse effects, opportunistic infections, secondary therapy failure of biological treatment, steroid induced acne, cushingoid reactions and steroid-dependent course of disease as relevant medication adverse effects.

Comparison of mean Z-scores for height and body mass index (BMI) at the age of 18 years represents growth outcome and nutritional status. Paediatric data were correlated with data of the normal reference population [23]. Z-Scores lower than − 2 implied growth retardation or underweight, respectively. A delay of sexual development was acknowledged if the respective physician stated this condition. Multiplication of average effective radiation doses caused by diagnostic imaging (computerised tomography of abdomen, barium enema, barium follow-through, abdominal and chest radiograph) with the number of investigations was used to calculate the cumulative radiation exposure [24].

Quality of Life (QoL) as an important clinical outcome parameter, was assessed by the German version of the validated IBD Questionnaire (IBDQ-32) [25]. Thirty-two items on a seven-point Likert Scale in four subscales (bowel function, systemic symptoms, emotional and social function) were included (total score 32 to 224 points). High total scores implicate good and low scores poor QoL [26, 27]. Another questionnaire was added to the IBDQ to evaluate recent smoking, educational and employment status, as well as adherence to medication on a 5-point Likert scale. Questionnaires were sent to patients via post and were also available online using EvaSyS version 7.1 (Electronic Paper Evaluation systems GmbH, Lüneburg, Germany). Results represent the QoL and the beforementioned socioeconomic values after the 4-year observation period. The survey was conducted between June 2016 and February 2017. The questionnaires were answered by transfer and transition group patients with a median interval of eight and three years after the first visit at the adult gastroenterological clinic, respectively.

We separated cost calculations for medical treatment with biologicals (adalimumab and infliximab), non-biologicals including corticosteroids (oral, systemic and locally active), topical and oral 5-aminosalicylic acid (5-ASA) and thiopurines (oral), nutritional therapy (exclusive enteral nutrition (EEN)), hospitalisation and surgery costs, as well as additional investigation costs (imaging and endoscopies) [28]. Investigations included upper gastrointestinal endoscopy, colonoscopy, abdominal ultrasound, magnetic resonance imaging (MRI), computerised tomography (CT), barium studies, and radiographs. Additional costs for antibiotics, dietary supplements, as well as for various blood and stool tests were not part of the cost analysis. The excluded expenditures had little relevance on the final cost analysis. In addition, consultations in the Day Care Unit setting (mainly performed for drug infusions) were excluded from clinical as well as economic outcome analyses because of individual financial agreements between the hospital and the health insurance companies.

We applied the German Uniform Assessment Standard (EBM, in its current version from April 2017) for the cost analyses of diagnostic investigations. We used German Diagnosis Related Groups (G-DRG, version 2017), Operation and Procedure Keys (OPS, version 2017) and the web-grouper provided by the University of Münster, Germany for hospitalisation and surgery cost calculation (drg.uni-muenster.de, G-DRG 2018). We obtained prices for standard medications and biologicals from the official German Pharmaceutical Catalogue (Red List, version 1.0.1, 2017). Treatment duration was calculated on a daily basis using the documented dates for the beginning and end of each drug treatment. The largest package of the cheapest generic drug of the respective agent was used to calculate drug expenses per day. All five cost factors were summarised to calculate total disease management expenditures within the study period and were converted into costs per year for better comparability with other cost evaluations.

### Statistical analysis

All data were analysed using SPSS statistical package 23.0 (SPSS Inc. Chicago, Illinois, USA). Descriptive statistics were generated for continuous and categorical variables. Statistics included median, range, mean and standard deviations (SD) of continuous variables, as well as frequencies and relative frequencies of categorical factors. Testing for differences of continuous variables between the study groups was accomplished by the 2-sample *t* test for independent samples or the Mann-Whitney U test, as appropriate. Test selection was based on evaluating the variables for normal distribution employing the Kolmogorov-Smirnov test. Comparisons between the study groups for categorical variables were performed using the chi-square test or the Fisher’s exact test. All *p* values resulted from two-sided statistical tests and values of *p* < 0.05 were considered statistically significant.

The response for each question in the IBDQ ranges from severe impairment (value of 1) to no impairment (value of 7). We summarised the items of the respective scale to calculate IBDQ subscale and total scores. If patients omitted a question, we handled this situation following the example set by the German validation study by Janke et al. (2005): A maximum number of two items in the IBDQ subscales for emotional and bowel function and one item in the evaluation of systemic symptoms and social function were allowed to be missing [26]. The absent items were then substituted by the mean of the respective subscale score. Nonetheless, no answered questionnaire had to be excluded due to a higher number of missing items in our study. Findings from the cost analyses were rounded to the nearest whole euro (€).

## Results

### Demographic and clinical characteristics

Mean age at diagnosis was 14 years for transfer patients (*n* = 11; SD: ±2 years) as well as for transition patients (*n* = 24; SD: ±3 years). The median age at transfer in the transfer group was 20 years (range 17 to 22 years) and 18 years in the transition group (range 17 to 21 years, *p* = 0.082). Disease presentation and prevalence of EIM did not significantly differ between the groups. Two transition (8%) and one transfer patient (9%) had an acute pancreatitis within the study period. 88% of patients in the transition group and 63% in the transfer group were in remission at their first referral to the adult gastroenterology clinic (*p* = 0.171). The transfer group includes IBD patients that where transferred before implementation of the transition program and the transition group includes adolescents with IBD that where transferred after implementation of the transition program. Therefore, the transfer group does not necessarily represent / families who were not fully engaged in their care or were probably not receiving optimal treatment. All demographic and clinical background data are summarised in Table 1.

**Table 1:** Demographic and clinical data

	Transfer (*n* = 11)	Transition (*n* = 24)	*p*-value
Gender male (M) / female (F), n (%)	M = 7 (64%) F = 4 (36%)	M = 14 (58%) F = 10 (42%)	1.000^*^
Diagnosis, n (%)			0.279^+^
CD	8 (73%)	12 (50%)	
UC	2 (18%)	11 (46%)	
IBDU	1 (9%)	1 (4%)	
Age at diagnosis in years	14 (±2)	14 (±3)	0.754^^^
Paris classification [20] of CD patients			
Age of onset, n (%)			0.209^+^
A1a	1 (12%)	0 (0%)	
A1b	10 (88%)	12 (100%)	
Location of disease, n (%)			0.082^+^
L1	2 (25%)	0 (0%)	
L2	0 (0%)	3 (25%)	
L3	6 (75%)	9 (75%)	
Upper gastrointestinal inflammation, n (%)			0.690^+^
L4a	4 (50%)	6 (50%)	
L4b	0 (0%)	1 (8%)	
Behaviour of disease at diagnosis, n (%)			0.402^+^
B1	8 (100%)	11 (92%)	
B2	0 (0%)	0 (0%)	
B3	0 (0%)	0 (0%)	
B2B3	0 (0%)	1 (8%)	
Perianal disease	3 (38%)	5 (42%)	1.000^*^
Growth			0.111^+^
G0	4 (50%)	10 (83%)	
G1	4 (50%)	2 (17%)	
Paris classification of UC patients			
Extension of inflammation, n (%)			0.701^+^
E1	0 (0%)	1 (9%)	
E2	0 (0%)	2 (18%)	
E3	0 (0%)	0 (0%)	
E4	2 (100%)	8 (73%)	
Disease severity, n (%)			0.140^+^
S0	1 (50%)	10 (91%)	
S1	1 (50%)	1 (9%)	
Total duration of follow-up in paediatric care in years (range)	5 (2–11)	3 (2–10)	0.303^#^
Age at transfer in years (range)	20 (18–22)	18 (17–21)	0.082^#^
Total duration of follow-up in adult care in years (range)	2 (2–8)	2 (2–5)	0.394^#^
Prevalence of previous and current EIM, n (%)	7 (64%)	13 (54%)	0.721^*^
Medical treatment before study period (at least used once), n (%)			
Thiopurines	11 (100%)	22 (92%)	1.000^*^
5-ASA	8 (73%)	18 (75%)	1.000^*^
Steroids	6 (55%)	16 (67%)	0.708^*^
EEN	3 (27%)	6 (25%)	1.000^*^
Biologicals	3 (27%)	5 (21%)	0.685^*^
Medical treatment within the study period (at least used once), n (%)			
Thiopurines	11 (100%)	22 (92%)	1.000^*^
5-ASA	8 (73%)	18 (75%)	1.000^*^
Steroids	8 (73%)	18 (75%)	1.000^*^
EEN	4 (36%)	6 (25%)	0.689^*^
Biologicals	5 (46%)	7 (29%)	0.451^*^

Legend. CD, Crohn’s disease; UC, ulcerative colitis; IBD, inflammatory bowel disease; IBDU, unclassified IBD; A1a, 0–< 10 years; A1b, 10–< 17 years; B1, non-stricturing-non-penetrating disease; B2, stricturing disease; B3, penetrating disease; B2B3, both penetrating and stricturing disease; G0, no growth failure; G1, evidence of growth delay [G1]; L1, distal 1/3 ileal disease (limited cecal disease); L2, colonic disease; L3, ileo-colonic disease; L4, upper gastrointestinal tract disease; L4a, esophago-gastro-duodenal disease proximal to ligament of Treitz; L4b, distal to ligament of Treitz; E1, ulcerative proctitis; E2, left-sided ulcerative colitis (distal to splenic flexure); E3, extensive colitis (hepatic flexure distally); E4, pancolitis (proximal to hepatic flexure); S0, not severe; S1, ever severe; extraintestinal manifestations, EIM; 5-ASA, 5-aminosalicylic acids; EEN, exclusive enteral nutrition; ^*^Fisher’s exact test; ^+^Chi-square test; ^#^Mann-Whitney U test; ^^^2-sample *t* test

### Clinical outcome values

Seven (64%) patients in the transfer group had an IBD-related intestinal complication within the study period compared to five (21%) patients in the transition group (*p* = 0.022). The number of surgical interventions during the study period tended to be lower in transition group patients compared to transfer group patients (13% vs. 46%, *p* = 0.077). There was a tendency towards a higher prevalence of abscesses in transfer group patients (27% vs. 4%, *p* = 0.082). In addition, bowel resections tended to be more frequent in the transfer group (1 ileocecal resection; 1 total colectomy), whereas no patient in the transition group needed bowel resection (18% vs. 0%, *p* = 0.092).

We found a significantly higher cumulated doses of diagnostic radiation exposure within the study period in the transfer group with a mean of 4.2 mSV (SD: ±5.3 mSv) compared to an average of 0.01 mSv (SD: ±0.01 mSv) in the transition group (*p* = 0.036). Two patients in the transfer group (18%) received an abdominal CT and two others (18%) received a barium contrast enema, while there were no patients in the transition group, who received such investigations (*p* = 0.092, respectively).

While the number of psychiatric diseases with onset during the study period did not vary between groups, disordered eating behaviour and classifiable eating disorders tended to appear more frequently in transfer group patients (18%), while no patient in the transition group was affected (p = 0.092). More specifically, one transfer patient was diagnosed with a hyporectic eating disorder, while the other one presented with long-lasting dysfunctional eating behaviour. Delayed puberty (stated by the paediatric gastroenterologist at date of transfer) was documented in three transfer group patients (27%), but was not reported in patients of the transition group (*p* = 0.025). Other clinical outcome variables including height and BMI z-scores did not significantly differ between transfer and the transition group. Table 2 provides further clinical details of the study population. Further details about the clinical outcome values are summarized in Additional file 1: Table S1.

**Table 2:** Clinical long-term parameters

	Transfer (*n* = 11)	Transition (*n* = 24)	*p*-value
Patients with at least one IBD-related intestinal complication, n (%)	7 (64%)	5 (21%)	0.022^*^
Patients with at least one IBD-related surgery, n (%)	5 (46%)	3 (13%)	0.077^*^
Number of IBD-related hospitalizations, n (range)	3 (0–11)	1 (0–3)	0.112^#^
Height Z-score at the age of eighteen			0.854^+^
-2 < Z, n (%)	1 (9%)	1 (4%)	
-2 ≤ Z ≤ 0, n (%)	6 (55%)	13 (54%)	
0 < Z ≤ 2, n (%)	4 (36%)	9 (38%)	
Z > 2, n (%)	0 (0%)	1 (4%)	
Height Z-score at the age of eighteen	−0.54 (±1.26)	−0.10 (±1.22)	0.328^^^
BMI Z-score at the age of eighteen			0.265^+^
-2 < Z, n (%)	2 (18%)	2 (8%)	
-2 ≤ Z ≤ 0, n (%)	7 (64%)	11 (46%)	
0 < Z ≤ 2, n (%)	2 (18%)	11 (46%)	
Z > 2, n (%)	0 (0%)	0 (0%)	
BMI Z-score at the age of eighteen	−0.56 (±1.22)	−0.25 (±1.02)	0.441^^^
Patients with documented pubertal delay, n (%)	3 (27%)	0 (0%)	0.025^*^
Patients with a psychiatric disorder diagnosed within the study period, n (%)	4 (36%)	3 (13%)	0.171^*^
Cumulated doses of radiation in mSv	4.2 (±5.3)	0.01 (±0.01)	0.036^#^
Patients with at least one adverse effect caused by IBD medication, n (%)	6 (55%)	12 (50%)	1.000^*^

Legend. IBD, inflammatory bowel disease; BMI, body mass index; mSV, millisieverts; ^*^Fisher’s exact test; ^+^Chi-square test; ^#^Mann-Whitney U test; ^^^2-sample *t* test

### Quality of life and nicotine consumption

IBDQ results and socioeconomic parameters are summarised in Table 3. Nine out of 11 (82%) transfer and 18 out of 24 (75%) transition group patients completed the survey (overall response rate 77%). There were no statistically relevant differences in IBDQ total or subscale scores between the two groups. Nevertheless, transition patients reported to be significantly less frequently limited in their sexual activity than patients in the transfer group (median scale value 7 (range 3 to 7) vs. 3 (range 1 to 7), *p* = 0.023) Transition patients also stated to have lower rates of loose bowel movements in the past two weeks compared to the transfer group (4 (range 1–7) vs. 5 (range 2–7), *p* = 0.053). IBDQ-32 answers for all items are summarized in Additional file 2: Table S2. There were no significant socioeconomic differences between the two groups (Table 3). None of the transfer group patients were active smokers, whereas seven patients in the transition group (39%) admitted active smoking (*p* = 0.059).

**Table 3:** IBDQ scores and socioeconomic parameters

	Transfer (*n* = 9/11)	Transition (*n* = 18/24)	p-value
IBDQ - Total score	158 (±39)	170 (±27)	0.329^^^
IBDQ - Emotional function	60 (±15)	60 (±12)	0.992^^^
IBDQ - Bowel symptoms	51 (32–69)	57 (39–65)	0.536^#^
IBDQ - Systemic symptoms	21 (±7)	25 (±6)	0.157^^^
IBDQ - Social function	24 (15–35)	33 (21–35)	0.146^#^
Current self-estimated adherence to medication on a 5-point Likert scale	4 (2–4)	3 (1–5)	0.940^#^
Active smoking	0 (0%)	7 (39%)	0.059^*^
High-school diploma	5 (56%)	10 (56%)	1.000^*^
Paid employment	6 (67%)	16 (89%)	0.295^*^

Legend. IBDQ, Inflammatory Bowel Disease Questionnaire; ^*^Fisher’s exact test; ^#^Mann-Whitney U test; ^^^2-sample *t* test. IBDQ scores and socioeconomic parameters are represented as median (range) and mean (±SD), respectively

### Expenditures in disease management

Table 4 summarises data from our treatment cost analyses. Annual medical costs including surgeries and hospital admissions tended to be lower in transition group patients (mean: 744 €, SD: ±630 €) compared to patients without transition support (mean: 2,691 €, SD: ±4,150 €) (*p* = 0.050). We found no statistical relevant differences between total treatment expenses or other cost parameters. Treatment with biologicals contributed an average amount of 50% (3,963 €) of mean total expenses in transfer group patients and 67% (4,301 €) in transition group patients.

**Table 4:** Distribution of treatment costs per year

	Transfer (*n* = 11)	Transition (n = 24)	p-value
Total expenditures	7,917 € (±7,766 €)	6,395 € (±8,768 €)	0.374^#^
Medical treatment with biologicals	3,963 € (±5,609 €)	4,301 € (±8,645 €)	0.529^#^
Surgery and hospitalization	2,691 € (±4,150 €)	744 € (±630 €)	0.050^#^
Medical treatment with non-biologicals	1,089 € (±392 €)	1,276 € (±567 €)	0.330^^^
Thiopurines	349 € (±149 €)	412 € (±159 €)	0.275^^^
5-ASA	694 € (±448 €)	836 € (±544 €)	0.252^#^
Steroids	45 € (±54 €)	28 € (±33 €)	0.655^#^
Exclusive enteral nutrition	109 € (±236 €)	38 € (±70 €)	0.476^#^
Additional outpatient investigations	66 € (±64 €)	37 € (±40 €)	0.192^#^

Legend. 5-ASA, 5-aminosalicylates; ^#^Mann-Whitney U test; ^^^2-sample *t* test. Data are represented as mean (±SD)

## Discussion

Although there is a number of expert recommendations [7, 29] and some outcome analyses in recent literature, the precise variables of an effective transition process still need to be clarified. One of the first transition outcome analyses for IBD patients found significant clinical improvements 2 years after transition support [15]. Lower surgery and hospitalisation rates, better clinic attendance rates, improved adherence to medication and reduced cumulative doses of radiation were reported benefits of the transition process. Bennett et al. (2016) published a survey from 46 IBD patients, previously treated in the paediatric clinic and transferred into adult care compared to a cohort of 35 IBD patients with a similar age, which were diagnosed before the age of 18, but were immediately followed up at an adult specialist clinic and thus did not went through a transition process. They also noted psychosocial outcome benefits and a positive perception of the transition process, but found neither an effect on clinical nor on psychosocial development [17]. After all, a survey by Dabadie et al. (2008) found that IBD patients and parents considered a structured transition in terms of a joint medical visit as highly beneficial for building confidence with the new adult gastroenterologist in almost every case [30]. Thus, transition care enables IBD patients to maintain the treatment success during paediatric care and on the other hand, empowers the physician-patient relations.

Prevalence of IBD-related intestinal complications was significantly lower in our transition group patients compared to IBD patients without transition support. In general, intestinal complications tend to be more frequent in IBD with paediatric onset [31, 32]. In addition, other studies showed that surgery and admission rates were significantly higher in IBD patients without transition support or lower in patients, who frequently attended transition clinics, compared to adult controls [15, 33]. This might result from lower rates of intestinal complications in post-transition patients and is therefore consistent with our findings. Furthermore, intestinal complications like perianal fistulas in CD patients do not only lead to higher health care utilisation rates, but are also associated with higher morbidity rates, impaired quality of life and relevant high expenses for following biological treatment [34, 35]. The prevention of intestinal complications by structured transition is thus of utmost importance for the clinical outcome of young adults with IBD, both, from a medical and economical point of view.

The cumulative radiation exposure during the study period was significantly lower in our transition group than in patients without transition support, which might be explained by the lower incidence of intestinal complications and subsequent surgery in the transition group. On the other hand, patients in the transition cohort were most commonly followed up by abdominal ultrasound and received only routine low-dose imaging studies such as chest x-rays before anti-Tumor Necrosis Factor alpha (anti-TNF) therapy. Disease course in IBD patients is commonly accompanied with high effective doses of radiation due to follow-up and diagnostic procedures over a lifetime [24], in particular in IBD patients with disease onset at an early age. A cumulated radiation exposure of more than 75 mSv already increased the risk of cancer related death by 7.3% in a cohort of 409 CD patients with an IBD diagnosis before the age of 17 years [36, 37].

Despite we observed a trend towards a lower prevalence of surgeries within the study period, the number of hospitalisations did not significantly differ between our transfer and the transition group. This is in contrast with findings from other IBD transition outcome studies. As already discussed by Cole et al. (2015), decreased admission rates in the transition period could result from optimised medication and adherence achieved by frequent alternating assessments by a paediatric and an adult medical team [15]. However, this approach was not a standardised part of the transition program used in our present study. Another explanation for our findings could be the methodology of the outcome analysis like the length of the reviewed follow-up period. Various studies also recommend the evaluation and optimisation of transition readiness before the final transfer date and its role to prevent early relapse or complications. Although we avoided to transfer patients with unstable disease, we did not utilise validated tools acknowledging clinical as well as socio-ecological factors to access transition readiness before the first visit at the adult clinic [38–40]. Furthermore, the impact of biological treatment on hospital admission and surgery rates should be taken into consideration [41, 42]. Reduction of surgery and hospitalisation rates is still an important target for transition support.

The overall incidence of psychiatric comorbidities showed no difference between our study groups, except for variance of newly diagnosed eating disorders and disordered eating behaviours. However, eating disorders like anorexia nervosa, but more importantly dysfunctional eating habits including both skipping meals and binge eating are common in IBD patients. Nutritional deficiencies as well as obesity are possible consequences of these psychological comorbidities and are potentially worsening IBD [43–45]. A 41% greater trend towards misuse of medication and excessive exercise to achieve a desired body image was described in patients with diet-related chronic health conditions such as type 1 diabetes, cystic fibrosis and IBD [46]. Preparation for transition should enlighten the consequences of non-adherence and inadequate diet as long as intervention is possible. Non-adherence to medication might be one of the most important issues in IBD care and is crucial for successful transition to the adult setting. Thirty to 45% of IBD patients are non-adherent, which increases costs and causes negative effects on disease outcome [47, 48]. Improved medication adherence in adolescents has been described for larger IBD clinics [33]. Adherence rates to medication (based on patients’ survey data) did not significantly differ between our two study groups and therapeutic drug monitoring was not systematically performed in our patients.

A significant higher number of transfer group patients had a documented history of delayed puberty at the time of transfer into adult health care compared to patients in the transition group. It should be noted, however, that sexual development data by Tanner staging was not systematically assessed in our patients like in other retrospective studies [49]. Body growth and weight outcome data showed no significant differences between our relatively small study groups during the transition period. However, it is known that about 20% of patients with childhood onset CD have a significantly reduced adult height in larger IBD cohorts [50, 51]. A higher BMI is also associated with a lower risk for surgery, clinical relapse and loss of response to anti-TNF therapy [52]. Although anthropometric outcome might not be directly affected by transition care, prevention of underweight and growth retardation is still an important task during the transition process.

Seven out of 24 transition patients (39%) stated active nicotine consumption compared to none in the transfer group. Forty-four percent of our UC patients were active smokers in contrast to 17% of our CD patients, whereas at the same time the number of UC patients was higher in our transition cohort (*n* = 11/24; 46%) compared to our transfer cohort (*n* = 2/11; 18%). Even though there is published evidence for beneficial effects of smoking in UC patients [53] we did not believe that this may have affected the clinical outcomes during our study period.

Educational and employment status evaluated after the 4-year observation period did not significantly vary between our transfer and transition group patients. Although young college students with IBD feel less successful and admitted their class attendance rates to be less regular than their healthy controls [54], IBD patients achieved a diploma or a post-secondary school degree more frequently than their healthy peers and earn more money per annum [55, 56]. Since time of transfer and graduation from school occur at a similar time, transition support may enable more patients to start into a successful work life as an adult.

QoL is known to be significantly reduced in CD [57] and UC patients [58] and improving QoL is a major task in the management of IBD patients. Sexual activity was significantly reduced in our transfer group patients compared to transition group patients. This is a common finding in IBD with a rate of up to 58% of patients stating impaired sexual function and libido [59]. Dissatisfaction with the body image is also present in approximately two-thirds of IBD patients and in up to half of cases, disease already had a negative influence on relationships [60]. Therefore, our IBD transition program addresses information about sexuality and fertility in preparation for transfer. Evaluation of a German Modular Patient Education Program (*‘ModuS’*) indicated a positive effect on patients’ self-efficacy and satisfaction [61]. Transition patients also tended to subjectively perceive their bowel movements to be less frequently loose than in the transfer group. Better control of intestinal symptoms, also considering the clinical outcomes, might explain this finding. Other domains of the IBDQ did not significantly differ between our study groups.

Total disease management costs during the transition process were another main outcome parameter of our study, but showed no significant differences between our groups. To our knowledge, there are little or no other published studies available, which evaluate the cost-effectiveness of structured transition programs. Importantly, one of the main cost-drivers in both our study groups was the use of biologicals. The implementation of transition programs might also have indirect long-term financial benefits since active disease in IBD patients is associated with high costs by sick leaves, occupational disability and early retirement [62, 63]. Long-term cost savings might result from prevention of disease relapses during the time of transition when young adults with IBD start their professional training / life. Overall, surgery and admission expenditures tended to be lower in the transition group. This may reflect the significant lower incidence of IBD-related complications and need of surgery in our transition group. One of the main ideas behind transition care is that imparting of disease-related knowledge and self-management skills in patients with chronic conditions prevents disease relapse or exacerbation. A patient education program with an estimated price of 200 US dollar per patient described in a study from the USA showed possible reimbursements in chronically ill patients by lower health care utilisation rates [64]. The randomised controlled trial by Kennedy et al. (2004) also demonstrated potential cost savings through better adherence, lower admission rates and improved quality of life in IBD patients one year after self-management education [65]. A validated 24-item questionnaire called CCKNOW (Crohn’s and Colitis Knowledge) examines disease-related knowledge about IBD, medication, diet and complications. Colombara et al. (2014) demonstrated possible reimbursements of about 1,100 € per an increase of 5 points on the CCKNOW scale [66]. Thus, we attribute the tendentially lower health care utilisation costs to educational core elements of our setting, which includes the improvement of disease-related knowledge, self-management skills and the evaluation of these education parameters at the joint consultation. Our finding that biologicals are not only the main cost drivers in both our study groups, but also have a significant impact on mean total disease management costs per year, is consistent with other cost analyses: 64% of total health care costs in CD and 31% in UC patients were distributed to anti-TNF therapy in a Dutch study, while surgery and hospitalisation expenditures, which were the main cost drivers before introduction of biological therapy in clinical practice, were much lower [67]. These findings might be explained by the lower health care utilisation rates caused by biological treatment in IBD [68]. For example, anti-TNF therapy alone leads to average savings of 2,750 pounds per IBD patient after one year in a study from the UK [69]. Further transition cost analyses should take the influence of anti-TNF therapy on total disease management costs into consideration. Finally, costs for the implementation of structured transition programs need to be considered but are most likely justified by the medical and economic (long-term) benefits of transition support.

The design of our present study might have some potential limitations. Since our retrospective single-centre study included a relatively small number of patients and the study objects were not randomised, most results have to be interpreted as trends and should be further evaluated in larger studies. We decided to use a retrospective study design and a historic patient cohort in this pilot study since our local ethics committee had serious ethical concerns regarding randomization between a simple transfer and structured transition into adult health care. In addition, participation in a structured transition program for adolescents with IBD in Germany is to a certain degree still dependent on a declaration from the health insurer on assumption of the costs. In addition, the outline of the transition process in our present study differs from other larger transition programs. New transition programs in Germany like the BTP, for example, include case managers, information about treatment procedures, attendance surveillance and specialised allied-health IBD professionals. On the other hand, some transition initiatives provide an even closer and more regular support of affected young adults with IBD. For example, a biweekly transition clinic including a paediatric and adult gastroenterologist, as well as an IBD nurse is held for IBD patients by the Barts and the London National Health Service (NHS) trust [33]. Other transition programs for IBD patients mainly aim to increase adherence to medication by utilisation of smartphone apps as a reminder for daily medication intake [70]. Future developments in transition medicine and larger studies may confirm and clarify the specific beneficial effects of standardised structured transition support and programs.

## Conclusions

Our study underpins the potential beneficial effects of structured transition care in adolescents with IBD. Implementing a structured transition program like the German BTP might provide a comprehensive transition from adolescent to adult care. This is of utmost importance since deficiencies in care during this particularly critical phase can result in treatment interruptions, insufficient treatment compliance and increased frequency of complications that are probably avoidable. However, the cost-benefit ratio in transition care is still a subject of controversial discussion, thus many German health insurances still do not financially support transition programs. Future large-scale multicentre studies with carefully selected control groups could help to support the findings of our single centre retrospective study and to increase the statistical impact.

## Additional files

Additional file 1: **Table S1.** Detailed clinical outcome values. (DOCX 15 kb)

Additional file 2: **Table S2.** IBDQ-32 answers for all items. (DOCX 18 kb)

## Data Availability

The datasets used and/or analysed during the current study are available from the corresponding author on reasonable request.

## Abbreviations

BMI: Body mass index
BTP: Berlin Transition Program
CCKNOW: Crohn’s and Colitis Knowledge
CD: Crohn’s disease
EIM: Extraintestinal manifestations
G-DRG: German Diagnosis Related Groups
IBD: Inflammatory bowel diseases
IBDQ: IBD Questionnaire
mSv: Millisieverts
QoL: Quality of Life
SD: Standard deviation
TNF: Tumor Necrosis Factor
UC: Ulcerative colitis
UK: United Kingdom
