# Supplementary material for: Long-term implications of structured transition of adolescents with inflammatory bowel disease into adult health care: a retrospective study
Source: BMC Gastroenterol. 2019 Jul 19;19:128. doi: 10.1186/s12876-019-1046-5 (PMC6642593; doi:10.1186/s12876-019-1046-5)
Supplement: Supplementary file 1 — Table S1. Detailed clinical outcome values. (DOCX 15 kb) [file 12876_2019_1046_MOESM1_ESM.docx]

**Supplementary Table 1.** Detailed clinical outcome values.

| n (%) | **Transfer**  **(n = 11)** | **Transition**  **(n = 24)** | **p-value** |
| --- | --- | --- | --- |
| **Patients with at least one IBD-related complication** | 7 (64%) | 5 (21%) | 0.022^*^ |
| Fistula | 3 (27%) | 2 (8%) | 0.297^*^ |
| Abscess | 3 (27%) | 1 (4%) | 0.082^*^ |
| Stenosis | 2 (18%) | 3 (13%) | 0.640^*^ |
| Dysplasia/neoplasia | 0 (0%) | 1 (4%) | 1.000^*^ |
| Need for any bowel resection | 2 (18%) | 0 (0%) | 0.092^*^ |
| **Patients with at least one IBD-related admission** | 9 (82%) | 18 (75%) | 1.000^*^ |
| Caused by suspected or actual relapse | 7 (64%) | 16 (67%) | 1.000^*^ |
| Caused by elective endoscopy | 4 (36%) | 7 (29%) | 0.709^*^ |
| Caused by surgery | 5 (46%) | 3 (13%) | 0.077^*^ |
| Caused by other reasons | 3 (27%) | 2 (8%) | 0.297^*^ |
| **Patients with at least one IBD-related surgery** | 5 (46%) | 3 (13%) | 0.077^*^ |
| Caused by IBD-related complication (fistula, abscess and stenosis) | 4 (36%) | 3 (13%) | 0.171^*^ |
| Caused by any bowel resection | 2 (18%) | 0 (0%) | 0.092^*^ |
| Caused by other reasons | 1 (9%) | 1 (4%) | 0.536^*^ |
| **Patients with a psychiatric disorder diagnosed within the study period** | 4 (36%) | 3 (13%) | 0.171^*^ |
| Depressive disorder | 2 (18%) | 1 (4%) | 0.227^*^ |
| Disordered eating behaviour and eating disorders | 2 (18%) | 0 (0%) | 0.092^*^ |
| Anxiety disorder | 2 (18%) | 1 (4%) | 0.227^*^ |
| Adaptive disorder | 1 (9%) | 1 (4%) | 0.536^*^ |
| **Patients with at least one adverse effect caused by IBD medication** | 6 (55%) | 12 (50%) | 1.000^*^ |
| Haematopoiesis disorder (thiopurines or 5-ASA) | 2 (18%) | 6 (25%) | 1.000^*^ |
| Polyneuropathy (thiopurines) | 1 (9%) | 0 (0%) | 0.314^*^ |
| Dermatological adverse effect (thiopurines or 5-ASA) | 2 (18%) | 4 (17%) | 1.000^*^ |
| Opportunistic infections (biologicals) | 2 (18%) | 2 (8%) | 0.575^*^ |
| Secondary therapy failure (biologicals) | 2 (18%) | 2 (8%) | 0.575^*^ |
| Steroid induced acne | 1 (9%) | 2 (8%) | 1.000^*^ |
| Cushingoid reaction | 2 (18%) | 1 (4%) | 0.227^*^ |
| Steroid-dependent disease | 2 (18%) | 1 (4%) | 0.227^*^ |
| **Patients examined with diagnostic radiation** | 6 (55%) | 8 (33%) | 0.283^*^ |
| Abdominal computerized tomography | 2 (18%) | 0 (0%) | 0.092^*^ |
| Barium enema | 2 (18%) | 0 (0%) | 0.092^*^ |
| Abdominal radiograph | 1 (9%) | 0 (0%) | 0.314^*^ |
| Chest radiograph | 5 (46%) | 8 (33%) | 0.708^*^ |
| Other | 2 (18%) | 0 (0%) | 0.092^*^ |

**Legend**. IBDQ, inflammatory bowel disease; 5-ASA, 5-Aminosalicylates; ^*^Fisher’s exact test.
