# Supplementary material for: Long-term implications of structured transition of adolescents with inflammatory bowel disease into adult health care: a retrospective study
Source: BMC Gastroenterol. 2019 Jul 19;19:128. doi: 10.1186/s12876-019-1046-5 (PMC6642593; doi:10.1186/s12876-019-1046-5)
Supplement: Supplementary file 2 — Table S2. IBDQ-32 answers for all items. (DOCX 18 kb) [file 12876_2019_1046_MOESM2_ESM.docx]

**Supplementary Table 2.** IBDQ-32 answers for all items.

|  | **Transfer**  **(n = 9/11)** | **Transition**  **(n = 18/24)** | **p-value** |
| --- | --- | --- | --- |
| **Bowel symptoms** |  |  |  |
| B1 - Frequency of bowel movements | 5 (2-7) | 6 (3-7) | 0.403^#^ |
| B5 - Stool consistency | 3 (1-7) | 5 (2-7) | 0.053^#^ |
| B9 - Abdominal cramps | 6 (3-7) | 7 (3-7) | 0.705^#^ |
| B13 - Abdominal pain | 6 (3-7) | 6 (3-7) | 0.668^#^ |
| B17 - Flatulence | 4 (3-7) | 5 (2-7) | 0.433^#^ |
| B20 - Feel of a bloated belly | 4 (2-7) | 6 (3-7) | 0.433^#^ |
| B22 - Rectal bleeding | 7 (1-7) | 7 (3-7) | 0.631^#^ |
| B24 - Empty belly, but still urge to defecate | 4 (3-7) | 6 (3-7) | 0.668^#^ |
| B26 - Grimed underwear | 7 (2-7) | 7 (3-7) | 0.820^#^ |
| B29 - Nausea | 7 (4-7) | 6 (2-7) | 0.463^#^ |
| B – Subscale Score | 51 (32-69) | 57 (39-65) | 0.561^#^ |
| **Emotional function** |  |  |  |
| E3 - Frustration/restlessness | 4 (2-6) | 4 /2-7) | 0.433^#^ |
| E7 - Worries about surgeries | 7 (4-7) | 6 (2-7) | 0.463^#^ |
| E11 - Fear of not finding toilets | 6 (3-7) | 7 (2-7) | 0.705^#^ |
| E15 - Prostration | 4 (2-7) | 5 (2-7) | 0.820^#^ |
| E19 - Fear of cancer/relapse | 7 (3-7) | 5 (2-7) | 0.253^#^ |
| E21 - Feel of being relaxed | 3 (1-7) | 3 (1-7) | 0.596^#^ |
| E23 - Embarrassment | 6 (3-7) | 6 (2-7) | 0.860^#^ |
| E25 - Feel of despair | 7 (2-7) | 7 (2-7) | 0.820^#^ |
| E27 - Annoyed about IBD | 4 (2-7) | 6 (3-7) | 0.523^#^ |
| E30 - Testiness | 4 (2-7) | 3 (2-7) | 0.348^#^ |
| E31 - Lack of sympathy from others | 6 (2-7) | 7 (3-7) | 0.527^#^ |
| E32 - General satisfaction with life | 4 (3-7) | 4 (1-7) | 0.743^#^ |
| E – Subscale Score | 60 (±15) | 60 (±12) | 0.992^^^ |
| **Social function** |  |  |  |
| So4 - Incapability of going to work/school | 6 (3-7) | 7 (3-7) | 0.705^#^ |
| So8 - Cancelling social contacts | 6 (2-7) | 7 (4-7) | 0.253^#^ |
| So12 - Problems in leizure activities/sports | 5 (2-7) | 6 (3-7) | 0.348^#^ |
| So16 - Avoiding events without toilets | 7 (3-7) | 7 (4-7) | 0.980^#^ |
| So28 - Limited sexual activity | 3 (1-7) | 7 (3-7) | 0.023^#^ |
| So – Subscale Score | 24 (15-35) | 33 (21-35) | 0.160^#^ |
| **Systemic symptoms** |  |  |  |
| Sys2 - Tiredness | 3 (1-5) | 4 (1-7) | 0.160^#^ |
| Sys6 - Energy | 4 (2-6) | 5 (2-7) | 0.194^#^ |
| Sys10 - Feel of discomfort | 5 (2-7) | 5 (3-7) | 0,705^#^ |
| Sys14 - Decreased sleep/rest | 4 (1-7) | 4 (3-7) | 0.561^#^ |
| Sys18 - Problems in maintaining weight | 5 (1-7) | 7 (2-7) | 0.194^#^ |
| Sys – Subscale Score | 21 (±7) | 25 (±6) | 0.157^^^ |
| **IBDQ total score** | 158 (±39) | 170 (±27) | 0.329^^^ |

**Legend**. B, bowel symptoms; E, emotional function; So, social function; Sys, systemic symptoms; ^#^Mann-Whitney U test; ^^^2-sample *t* test. Data are represented as median (range) and mean (±SD), respectively.
